# HPV-specific innovations and controversies in therapeutic strategies for HPV-associated oropharyngeal carcinoma

**DOI:** 10.3389/fimmu.2026.1818274

**Published:** 2026-06-01

**Authors:** Yulun He, Jiaqi Tang, Yun Li, Yating Hu, Yaodong He, Xiaolong Zhang, Yanbing Yao, Jiale Wang, Kunyu Chen, Yuqi Wang, Huan Li, Jianhua Wei

**Affiliations:** State Key Laboratory of Oral & Maxillofacial Reconstruction and Regeneration, National Clinical Research Center for Oral Diseases, Shaanxi Key Laboratory of Stomatology, Department of Oral and Maxillofacial Surgery, School of Stomatology, The Fourth Military Medical University, Xi’an, Shaanxi, China

**Keywords:** HPV-associated oropharyngeal carcinoma, de-escalation therapy, liquid biopsy, immunotherapy, targeted therapy

## Abstract

The global surge in human papillomavirus (HPV)-associated oropharyngeal squamous cell carcinoma (OPSCC) has necessitated a fundamental shift in clinical management. While patients exhibit favorable prognoses compared to HPV-negative counterparts, balancing oncological cure rates with toxicity reduction remains a critical challenge. This review elucidates the virological mechanisms driven by oncoproteins E6 and E7 and evaluates novel diagnostic biomarkers, emphasizing the utility of liquid biopsy (circulating tumor HPV DNA,ctHPV DNA) and artificial intelligence for dynamic risk stratification. We critically examine current therapeutic controversies, particularly the risks of recurrence associated with unguided treatment de-escalation. Furthermore, the article highlights innovations in precision medicine, including immune reactivation strategies leveraging viral antigens (therapeutic vaccines, adoptive cell therapy) and targeted interventions exploiting metabolic vulnerabilities (PI3K/Akt/mTOR pathway) and DNA damage repair defects. Ultimately, we advocate transitioning from static TNM staging to a multidimensional, adaptive treatment model integrating genomics and immunology to achieve precise, individualized care for HPV-associated OPSCC.

## 1 Introduction

The epidemiological landscape of head and neck squamous cell carcinoma (HNSCC) has fundamentally shifted. While tobacco- and alcohol-related HNSCCs are declining, human papillomavirus (HPV)-associated oropharyngeal squamous cell carcinoma (OPSCC) incidence is growing explosively worldwide, particularly in Western countries (1).A U.S. survey and recent meta-analysis through 2025 indicate that the proportion of HPV-positive oropharyngeal cancer in the United States has surged from <16.3% in the 1980s to 73.8% in the 2020s (2, 3).Compared to traditional HPV-negative HNSCC, HPV-positive OPSCC exhibits unique biological behaviors and clinical characteristics. These patients are highly sensitive to chemoradiotherapy and demonstrate a significantly superior prognosis, achieving a 3-year overall survival(OS) rate of 82.4% (4).Recognizing this distinct survival advantage, international organizations introduced a separate staging system for HPV-positive oropharyngeal cancer in 2016, formalizing its status as an independent disease entity (5).

With prolonged survival, the long-term toxicities of conventional chemoradiotherapy have become increasingly prominent, severely compromising quality of life. Consequently, “therapeutic de-escalation”—reducing treatment intensity while preserving efficacy—has emerged as a major clinical research focus. However, unguided de-escalation may increase recurrence risks in certain high-risk cohorts, and current practice lacks robust risk stratification tools for individualized treatment. Meanwhile, emerging technologies like liquid biopsy and artificial intelligence-assisted diagnosis offer novel avenues for dynamic disease monitoring and early recurrence warning.

Crucially, the distinct viral-driven oncogenesis and the inherently active immune microenvironment of HPV-positive tumors establish a robust biological foundation for emerging precision interventions, including immunotherapeutic and targeted strategies (6, 7). Building upon this premise, this review systematically elucidates the virological basis and epidemiological characteristics of HPV-positive OPSCC, focusing on the application value of novel biomarkers like liquid biopsy in diagnostic stratification. Simultaneously, we comprehensively review innovations and controversies in therapeutic strategies, encompassing de-escalation indications, immunotherapy and targeted therapy advancements, and breakthroughs in prophylactic and therapeutic vaccines. The ultimate objective is to provide theoretical evidence and clinical references to achieve precise, individualized management of HPV-positive OPSCC.

## 2 HPV virology basis

### 2.1 HPV life cycle and genotyping

**Figure 1:**
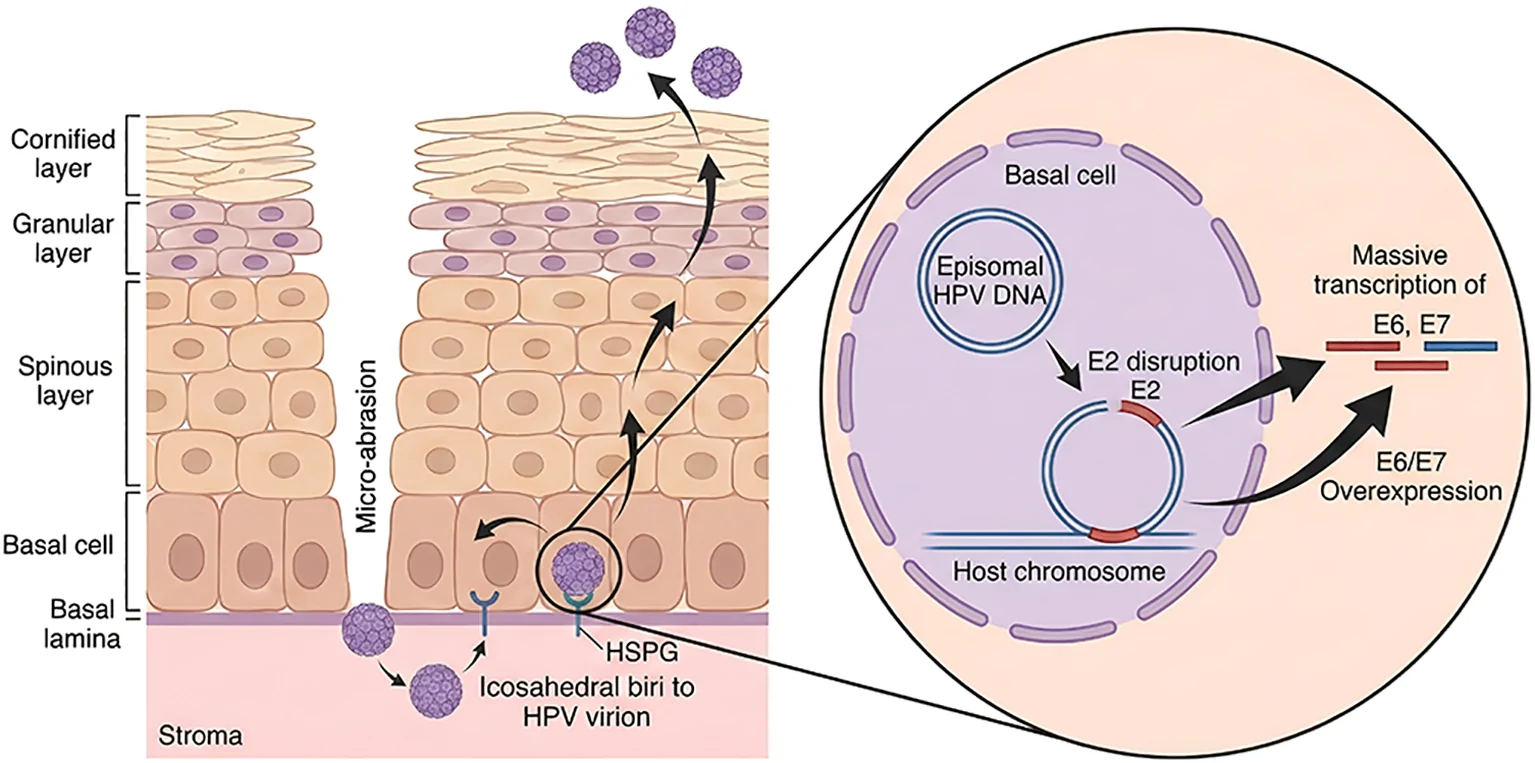
The life cycle of human papillomavirus and the molecular mechanism of genomic integration-driven carcinogenesis. The left panel illustrates the infection pathway within the stratified squamous epithelium. HPV virions exploit micro-abrasions to access and infect undifferentiated basal cells, a process initiated by the binding of the L1 capsid protein to cell surface heparan sulfate proteoglycans (HSPG), which triggers conformational changes necessary for viral entry. The viral life cycle, including genome replication and virion assembly, is tightly coupled with the differentiation program of the host epithelial cells as they migrate from the basal to the cornified layers. The right panel details the critical transition from infection to malignant transformation within a basal cell. While the viral genome initially exists as an episome, accidental integration into the host chromosome during episodes of genomic instability frequently results in the disruption of the viral E2 gene. This integration event abrogates E2-mediated transcriptional repression, leading to the constitutive overexpression of the oncoproteins E6 and E7, which is a hallmark of HPV-driven tumorigenesis.

HPV is a non-enveloped, double-stranded DNA virus (~8 kb) comprising early (E) and late (L) gene regions (8, 9).HPV initially infects undifferentiated basal epithelial cells via micro-abrasions (**Figure 1**). The normal viral life cycle strictly depends on host epithelial differentiation and proceeds through six established stages: adsorption, entry, uncoating, replication, maturation, and release (8).Specifically, L1 proteins mediate initial receptor binding, L2 facilitates uncoating and nuclear entry, while E1 and E2 coordinately regulate early viral replication and episomal maintenance during host cell division. Crucially, malignant transformation in HPV-driven OPSCC deviates from this normal productive cycle. This transition stems from the accidental integration of the episomal HPV genome into host chromosomes during episodes of genomic instability, which represents a stochastic but critical event in malignant transformation (8–10).

Based on carcinogenic risk, HPVs are classified into high- and low-risk types (11). High-risk strains, predominantly HPV16 and 18 (12), possess specific genomic variants that further influence oncogenic potential (13), thereby uniquely driving epithelial cells into the cell cycle as the primary drivers of OPSCC.

### 2.2 Functions of viral oncoproteins

High-risk HPV genome integration into host chromosomes is a critical carcinogenic step. This event frequently disrupts the viral E2 gene, relieving its inhibition of E6 and E7, thereby promoting sustained oncoprotein expression (8, 14). The E6 protein binds cellular ubiquitin ligase E6AP, forming a trimeric E6/E6AP/p53 complex (15). This triggers the degradation of the p53 tumor suppressor, dismantling cell cycle regulation and DNA damage repair mechanisms. Concurrently, the E7 protein binds and inactivates the retinoblastoma protein (pRb), releasing E2F transcription factors to force the cell cycle into the S phase (14, 16, 17). Viral integration also triggers host genomic structural variations, such as copy number abnormalities and exon skipping, suppressing tumor suppressor genes while promoting oncogene expression (17).Furthermore, E6 and E7 exacerbate glutamine decomposition to reprogram metabolism, heavily promoting a tumor phenotype (18).

### 2.3 Mechanisms of HPV immune evasion

HPV employs sophisticated immune evasion strategies, including interfering with MHC-mediated antigen presentation, inhibiting T-cell function, and modulating inflammatory responses and antigen-presenting cells (19). Alpha-type HPVs rely on E5, E6, and E7 to activate the cell cycle during epithelial differentiation, creating a high-replication environment for optimal viral amplification and packaging (10). Crucially, the non-lytic nature of HPV results in a distinct lack of pro-inflammatory signals; consequently, host dendritic cells (DCs) remain unactivated and fail to infiltrate the local environment (20). These combined mechanisms guarantee persistent infection, cultivating a permissive microenvironment for carcinogenesis.

## 3 Epidemiology and risk factors

### 3.1 Global incidence trends and population characteristics

The incidence of HPV-positive oropharyngeal cancer exhibits pronounced disparities across regions, sexes, ages, and races (21–23).U.S. surveillance data indicate HPV-positive OPSCC incidence surged 225% from 1984 to 2004, while HPV-negative cases declined by 50%, suggesting HPV has emerged as an increasingly important pathogenic driver of OPSCC in the United States (2). Similarly, an Italian study reported HPV-driven oropharyngeal cancer proportions rising from 12% to 50% between 2000 and 2022, notably in the tonsil and tongue base (24).A meta-analysis of 101,574 patients showed no significant OS difference between sexes in U.S. cohorts, whereas international studies report superior OS for female patients (25).

### 3.2 Sexual Behavior Patterns and HPV Transmission Mechanisms

HPV transmission in head and neck cancer is heavily linked to sexual behavior. It has been proposed that because viral loads in female genital mucosa exceed those in male genital mucosa and skin, men performing oral sex may be exposed to higher viral doses. Decreasing ages of sexual debut and increasing partner counts over recent decades may have contributed to oral/oropharyngeal HPV exposure (26).Interestingly, comparisons across sexual orientations reveal complex and nuanced patterns driven more by specific behavioral risk factors and sexual network dynamics than by sexual identity alone. Recent epidemiological analyses emphasize that among men who have sex with men (MSM), the risk of high-risk oral HPV infection is heavily influenced by a higher number of recent oral sexual partners and concurrent HIV infection, which profoundly amplifies viral persistence (27). Furthermore, bisexual women and women who have sex with women (WSW) frequently demonstrate higher oral HPV prevalence compared to exclusively heterosexual women, a disparity highlighting the critical role of diverse mucosal exposures within specific sexual networks (23).These foundational observations have been rigorously validated by updated systematic reviews, further reinforcing how network-specific dynamics drive oral HPV transmission (28).

## 4 Diagnosis and biomarkers

### 4.1 Clinical standards and challenges of HPV detection

p16 immunohistochemistry (IHC) remains the most common clinical surrogate for HPV-positive OPSCC. However, p16^INK4a^ overexpression also occurs in some HPV-negative OPSCCs, posing false-positive risks (29, 30). Moreover, in low-HPV-prevalence regions, p16 positivity drops to 16.88%, limiting its standalone reliability (31). To enhance diagnostic accuracy, combining p16 IHC with HPV DNA/RNA ISH or E6/E7 RT-PCR is increasingly recommended (17, 32). Nevertheless, these combinatorial methods are costly and demand stringent sample quality, restricting application in resource-limited settings.

### 4.2 Liquid biopsy and dynamic monitoring​

The superior prognosis of HPV-positive OPSCC creates an urgent clinical need for treatment de-escalation; however, traditional staging and clinical risk factors lack the precision to safely guide individualized de-intensification (33, 34). HPV-positive tumors release circulating tumor HPV DNA (ctHPV DNA), which is detectable at diagnosis with high sensitivity and specificity. Crucially, transient oral HPV infections do not yield detectable ctHPV DNA (35).

Liquid biopsies targeting ctHPV DNA—which broadly detects fragmented viral genomes released by apoptotic or necrotic tumor cells—and tumor-tissue-modified viral (TTMV)-HPV DNA—a specialized metric capturing ultra-short viral DNA fragments with structural signatures unique to oncogenesis—enable highly accurate surveillance. Chera et al. (36) prospectively demonstrated that two consecutive positive post-treatment ctHPV DNA tests predicted recurrence with a 94% positive predictive value and 100% negative predictive value, offering early warnings 3.9 months prior to biopsy (36)and ~70 days before traditional imaging (37). Reflecting a broad consensus across multiple independent studies (38–42),ctHPV/TTMV-HPV DNA diagnostic sensitivity ranges from 84%–100%, with 94.4%–100% specificity. For recurrence surveillance, sensitivity is reported at 88.4%–100%, and specificity at 98.4%–100% (38, 41), although these metrics vary depending on the detection method used and the disease stage.

Furthermore, circulating cell-free HPV16 DNA (cfHPV16 DNA) acts as an independent prognostic factor for progression-free survival, identifying treatment failure earlier than imaging (43). Notably, ctHPV16-DNA is an independent prognostic factor for HPV-positive OPSCC and can serve as a prognostic tool for progression-free survival, providing biomarker support for individualized prognostic stratification (44). To detect ultra-low ctHPV DNA levels, HPV whole-genome sequencing (WGS) liquid biopsies have achieved 98.7% sensitivity and specificity, representing a highly promising non-invasive tool (45).Although standardization is pending, integrating liquid biopsies with clinical and radiological data will profoundly optimize OPSCC management.

### 4.3 Artificial intelligence-assisted diagnosis

Artificial intelligence (AI) and deep learning demonstrate significant potential across distinct clinical applications in OPSCC, spanning non-invasive HPV status prediction, early disease detection, and prognostic stratification. To clarify these diverse utilities, **Table 1** categorizes recent breakthroughs by their functional domains.

**Table 1:** Applications of artificial intelligence and machine learning in HPV-associated OPSCC.

Application domain	AI modality/Methodology	Model performance	Core clinical finding	Study characteristics	Reference
Early Disease Detection	LASSO regression classifier on genome-wide methylation profiling data	AUC = 0.970 (training set),AUC = 0.935 (internal validation set, bootstrap resampling)	Combined plasma EPB41L3 CpG methylation sites and oral HPV16 status achieved high accuracy for early OPSCC detection.	Prospective, single-center, n = 197	(46)
Non-invasive HPV Status Prediction	C3D 3D convolutional neural network pre-trained on the Sports-1M dataset	AUC = 0.81 (independent external validation)	CT-based deep learning model accurately distinguished HPV-positive and HPV-negative primary OPSCC without invasive biopsy.	Retrospective, multi-center, n = 850	(47)
Multimodal HPV Status Prediction	Logistic regression combining T1C MRI radiomics (77 features) and 6 clinical variables	AUC = 0.871 (independent test set, bootstrap resampling)	Multimodal model significantly outperformed single-modality models; smoking, higher T-stage (T3/T4), larger tumor size, less round shape, and heterogeneous texture were associated with HPV-negative tumors.	Retrospective, single-center, n = 153	(48)
Histopathology-based HPV Status Prediction	Supervised deep learning on routine H&E-stained whole-slide images	AUC = 0.80 (independent external validation)	The deep learning model (AUC = 0.80) outperformed the median diagnostic accuracy of experienced pathologists (AUC = 0.74) and yielded more consistent results.	Retrospective, multi-center, n = 273	(49)
Prognostic Stratification & TME Analysis	Deep learning on H&E-stained whole-slide images	AUC = 0.895 (internal validation),AUC = 0.780 (independent external validation)	Generated a “Digital-HPV score” for survival stratification; identified distinct immune microenvironment patterns associated with HPV status and prognosis.	Retrospective, multi-center, n = 481	(50)

Beyond initial prognostic models, the clinical applicability of AI is rapidly expanding toward real-time decision support and personalized treatment planning. These multimodal AI approaches hold immense promise to bridge traditional diagnostic gaps— such as the inherent subjectivity in standard histopathological grading and the inability of conventional imaging to delineate complex immune microenvironment patterns.

Furthermore, recent comprehensive evidence has heavily reinforced this clinical utility. A scoping review by Migliorelli et al. (51) highlighted that AI models analyzing routine H&E-stained digital histopathology can achieve over 90% accuracy in predicting HPV status, rivaling experienced pathologists and bypassing complex molecular assays (51). Similarly, a meta-analysis by Vos et al. (52) pooled data across multiple radiomics studies, demonstrating a robust combined AUC of 0.764 for non-invasive HPV prediction utilizing standard-of-care baseline imaging, thereby mitigating the morbidity associated with invasive biopsies (52).

Within specific studies, Lang et al. (47) utilized a CT-based 3D convolutional neural network to distinguish HPV statuses, achieving an AUC of 0.81 (47). Similarly, Bos et al. (48) demonstrated that combining MRI-based radiomics with clinical variables significantly boosted predictive performance, yielding an AUC of 0.871 (48). Additionally, genome-wide methylation profiling revealed that combining plasma EPB41L3 CpG sites with oral HPV16 status predicts early OPSCC with an AUC of 0.97 (46). In the realm of digital pathology, Klein et al. proved that deep learning models analyzing routine H&E specimens (AUC = 0.80) could outperform the median diagnostic accuracy of experienced anatomic pathologists (49). Furthermore, Wang et al. (50) applied deep learning to H&E-stained whole-slide images, generating a “Digital-HPV score” that statistically stratified patients by overall and disease-specific survival while highlighting distinct immune patterns, such as higher B/T-cell and lower macrophage levels in HPV-positive tumors (50).

However, despite its transformative potential, the clinical deployment of AI in OPSCC faces substantial limitations. The “black box” nature of deep learning algorithms often lacks the interpretability required for clinical trust. As extensively appraised by Vos et al. (52), current radiomics studies exhibit significant methodological heterogeneity, with Radiomics Quality Scores (RQS) varying widely—ranging from 1 to 22 out of 36—due to the lack of open-access data and unstandardized segmentation protocols. Moreover, algorithm performance remains highly scanner-dependent and susceptible to tissue heterogeneity. Therefore, extensive prospective, multi-center validation, alongside the development of explainable AI (XAI) frameworks, is strictly required before these tools can be routinely integrated into clinical workflows.

The diagnostic paradigm for HPV-positive OPSCC is rapidly shifting from static, single-marker histopathology toward dynamic, multimodal surveillance. While liquid biopsies and AI-driven algorithms offer unprecedented precision in tracking minimal residual disease (MRD) and non-invasively predicting tumor status, their routine clinical integration remains highly debated. The absolute thresholds for clinical intervention based on ctDNA clearance kinetics currently lack validation from large-scale prospective trials. Furthermore, clinical trust in AI models is severely hampered by their uninterpretable nature, methodological heterogeneity, and scanner-dependency. Resolving these standardization barriers and developing explainable frameworks are mandatory before these transformative tools can safely dictate personalized treatment de-escalation.

## 5 HPV-specific innovations and controversies in therapeutic strategies

### 5.1 De-escalation therapy: biological basis and controversies in precision decision-making

Approximately 30%–40% of HPV-positive OPSCC patients can undergo treatment de-escalation without compromising efficacy, profoundly reducing the acute and lifelong toxicities of conventional chemoradiotherapy (53). Current de-escalation strategies encompass four main approaches: substituting cisplatin with agents like cetuximab, employing induction chemotherapy followed by sequential therapy, utilizing standard or reduced-dose primary radiotherapy, and implementing transoral minimally invasive surgery (54–57).The American Society for Radiation Oncology (ASTRO) guidelines strongly emphasize balancing efficacy with toxicity via optimized dose fractionation, intensity-modulated radiation therapy (IMRT) for normal tissue sparing, and tailored concurrent systemic agents (58).

HPV-positive OPSCC’s heightened radiosensitivity primarily stems from viral E6/E7 oncoproteins dismantling host DNA repair mechanisms. Even in HPV-negative OPSCC, E6 transfection impairs DNA repair and enhances radiosensitivity (59). Furthermore, unspliced E6 transcripts produce E6*I, which promotes post-radiation apoptosis via prolonged p53-independent G2/M arrest (60), and GA-OH, a small-molecule inhibitor of HPV E6, has been shown to enhance the cytotoxicity of radiotherapy (61). Interestingly, EGFR activation impairs DNA damage repair, downregulates E6, and induces p53 to cause radioresistance; thus, cetuximab (an EGFR inhibitor) has been utilized in de-escalation trials to minimize the cytotoxicity of concurrent chemotherapy (62).

Multiple prospective studies validate de-escalation. The University of Chicago’s Phase 2 trial utilizing de-intensified (chemo)radiotherapy reported excellent 5-year OS and PFS rates of 90% (63). Hypofractionated radiotherapy regimens (e.g., 70 Gy in 33 fractions) have reduced grade ≥3 acute toxicities to 21% without sacrificing survival benefits (64). Transoral robotic surgery (TORS) facilitates precise resection and accurate pathological staging, heavily supporting surgical de-escalation (55). The prospective MC1273 and MC1675 trials confirmed TORS was associated with lower morbidity in HPV-positive patients (65). Furthermore, the ECOG-ACRIN E3311 trial demonstrated that for intermediate-risk patients, primary transoral surgery followed by reduced-dose postoperative radiotherapy achieved excellent oncological and functional outcomes (66).

Despite these successes, de-escalation remains controversial and should not be widely adopted outside clinical trials, as it risks suboptimal treatment (53, 67, 68). A comprehensive meta-analysis of 48 studies (38,929 patients) revealed that, compared to standard care, de-intensified treatment significantly reduced OS ((hazard ratio [HR] = 1.33)), PFS (HR = 2.11), locoregional control (HR = 2.51), and distant metastasis control (HR = 1.9) (69). Specifically, the De-ESCALaTE trial demonstrated that substituting cisplatin with cetuximab in low-risk patients provided no toxicity benefits but significantly compromised tumor control (68).

Because clinicians currently lack universally recognized methods to reliably predict de-escalation benefits, multimodal decision models are imperative. Zeng et (70). formulated the UW03 immune score to classify patients into distinct immune categories, successfully identifying cohorts sensitive to de-escalation (70). Sandulache et (53). argued that traditional static risk stratification ignores treatment effects, advocating for adaptive risk stratification frameworks to safely guide individualized de-escalation decisions (53).

The fundamental controversy in de-escalation therapy is the profound inadequacy of static clinical staging for precise patient selection. As evidenced by survival detriments in major Phase III trials, “blind subtraction” of therapies without predictive biomarkers inevitably risks undertreatment and compromised oncological control. Until dynamic, multimodal decision frameworks—integrating immune scores and adaptive treatment responses—are universally validated, de-escalation must remain strictly confined to rigorous clinical trials.

### 5.2 Immunotherapy: precision breakthroughs driven by tumor antigens

Retrospective biomarker analysis utilizing the PD-L1 Combined Positive Score (CPS) indicates that approximately 73.2% of HPV-positive OPSCC patients meet the criteria for FDA-approved PD-1 inhibitor therapy, identifying them as potential candidates for immunotherapy (71). Emerging strategies include therapeutic vaccines (with/without anti-PD-L1 adjuvants), peptide-HLA platforms, and adoptive cell therapies like tumor-infiltrating lymphocytes (TILs), TCR-T, and CAR-T (72). The unique immunotherapeutic advantage of HPV-positive OPSCC relies on viral E6/E7 acting as highly specific tumor antigens, which are continuously expressed in tumor cells but absent in normal tissues. For example, a first-in-human Phase 1 trial (NCT02858310) demonstrated that TCR-engineered T cells targeting HPV16 E7 successfully mediated regression in metastatic HPV-16-associated epithelial cancers—a landmark finding, though the cohort was not exclusively limited to OPSCC (73).

**Figure 2:**
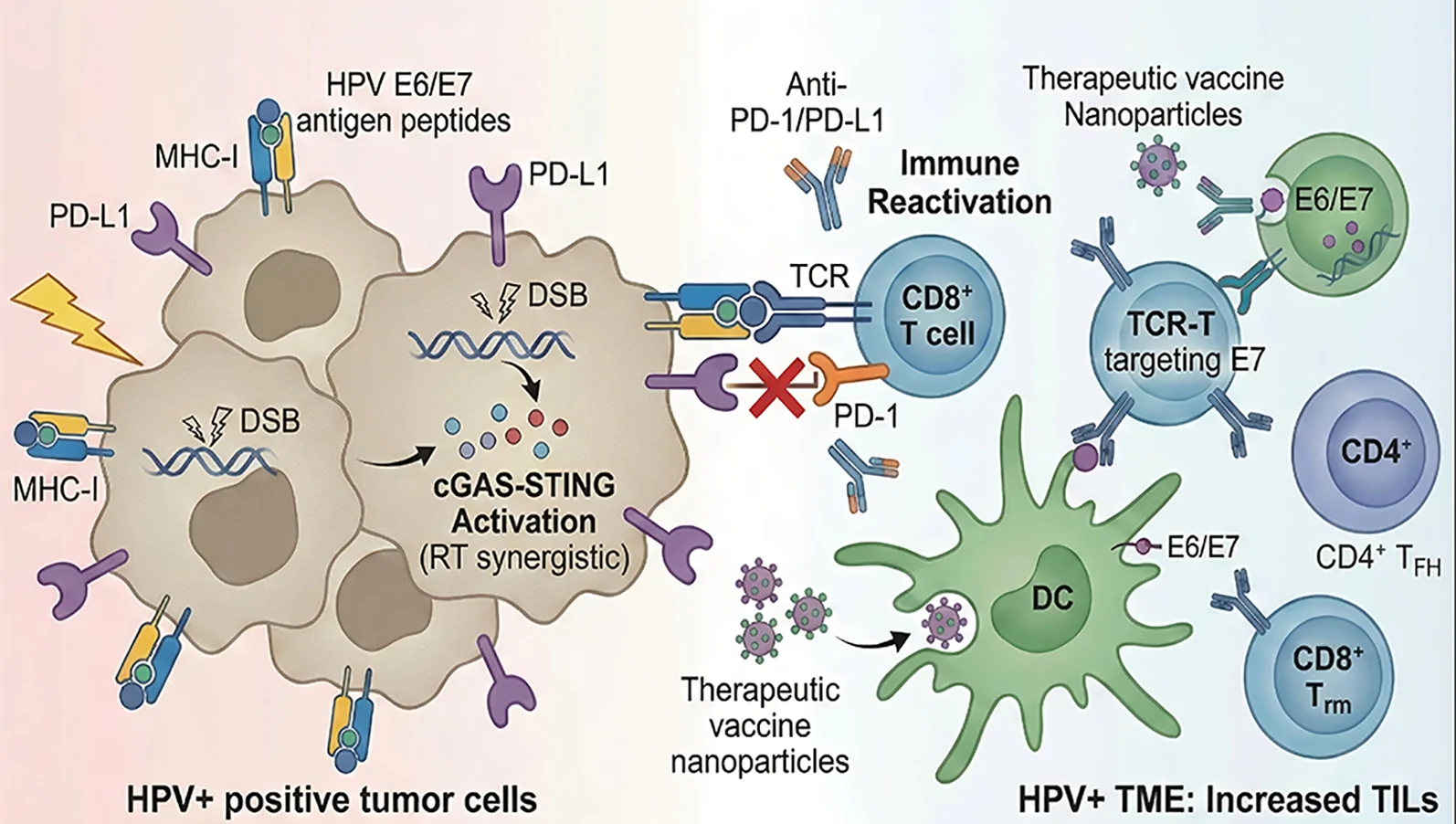
Immune microenvironment characteristics and therapeutic strategies in HPV-positive OPSCC. This figure illustrates the “virus-host” interaction landscape and targeted immunotherapeutic approaches. Left (Tumor Cell & Signaling): HPV-positive tumor cells continuously express viral oncoproteins E6 and E7, which are processed into antigen peptides and presented via MHC-I molecules, serving as specific targets. These tumors often exhibit elevated PD-L1 expression and defects in DNA repair (DSB), which can activate the cGAS-STING pathway—a mechanism that synergizes with radiotherapy to enhance immunogenicity. Middle (Immune Reactivation): ICIs (Anti-PD-1/PD-L1) block the inhibitory axis, reactivating CD8+ T cells to recognize and eliminate tumor cells. Right (Novel Strategies): Innovations leverage viral antigens for precision therapy. Therapeutic vaccines (e.g., nanoparticles) are uptake by DCs to prime HPV-specific CD4+ follicular helper T cells (TFH) and CD8+ resident memory T cells (Trm). Adoptive cell therapies, such as TCR-engineered T cells (TCR-T), are designed to specifically target the E7 antigen. The overall microenvironment is characterized by increased TILs, classifying it as an immunologically “hot” tumor.

Targeting strategies, by maintaining sustained antigen stimulation, create conditions for the immune system to precisely recognize and eliminate tumor cells, laying the molecular foundation for clinical translation. Immune checkpoint inhibitors (ICIs) have demonstrated stratified efficacy in the treatment of HPV-positive OPSCC (**Figure 2**). HPV-positive OPSCC patients have a significantly better prognosis driven by an active immune microenvironment. In these tumors, PD-L1+ cells and macrophages are located in closer spatial proximity to PD-1+ cytotoxic T lymphocytes (CTLs) (74), and this elevated PD-L1 expression is consistently associated with improved survival (75, 76). This upregulation extends across multiple immune checkpoint molecules (ICMs)—including CD27, PD-1, OX-40, and BTLA—which are significantly higher on TILs in HPV-positive compared to HPV-negative OPSCC (77). Crucially, this robust expression of immune checkpoints does not indicate immune exhaustion; rather, it reflects a sustained anti-tumor immune response linked to genetic variation burden and CTL enrichment (71). Furthermore, characterizing the specific functional states and spatial distribution of these immune cells is critical. Extensive literature confirms that robust populations of TILs strongly predict favorable clinical outcomes (78). Specifically, high levels of stromal CD8+ T-cell infiltration are significantly associated with improved survival (79). Beyond mere infiltration, the functional phenotype is paramount; recent evidence highlights that CD103+ tissue-resident memory T-cells (Trm) within the tumor microenvironment are the primary drivers of this potent anti-tumor immunity and prolonged patient survival (80). Accordingly, for treatment-naïve HPV-positive OPSCC patients, the combined application of immune checkpoint inhibitors with locoregional therapy has a theoretical basis (74). Preclinically, targeting interferon signaling with STING agonists combined with CTLA-4 blockade significantly amplified anti-PD-1 efficacy, driving robust tumor regression (81). Notably, single-cell sequencing identified functional PD-1+TCF-1+ stem-like CD8+ T cells in HPV-positive tumors; upon antigen stimulation, these cells differentiate into effector T cells and infiltrate tumors, dictating the anti-PD-1 response (82).

Previous cellular immunotherapies against HPV mostly focused on the E6 and E7 proteins of HPV-16; however, studies have found that HPV-16 specific T cells in HPV-positive OPSCC patients react not only to E6 and E7 but also recognize additional HPV proteins such as E1, E2, E4, E5, and L1. Simultaneously, treatment status was found to have the greatest impact on T-cell immunity: after patients received radical treatment (such as chemoradiotherapy), the variety of antigens recognized by HPV-specific T cells and the magnitude of the immune response were reduced compared to pre-treatment levels (83), accompanied by immunosuppressive effects such as increased numbers of regulatory T cells and a higher proportion of myeloid-derived suppressor cells (84). Furthermore, tobacco use significantly blunts T-cell infiltration, explaining the poorer prognosis of smokers with HPV-positive OPSCC (85). Conversely, neoadjuvant chemotherapy can actively enhance the immune response of HPV-specific TILs (86, 87).

The defining therapeutic advantage of HPV-positive OPSCC lies in viral non-self antigens (E6/E7) that drive a highly active, prognostically favorable immune microenvironment. However, a critical therapeutic paradox remains: conventional radical chemoradiotherapy profoundly depletes this antigen-specific T-cell repertoire and induces an immunosuppressive state. Furthermore, the narrow restriction of current adoptive cell therapies to single epitopes fails to capture the broad multi-antigen reactivity inherent to natural anti-tumor immunity, significantly limiting their universal clinical efficacy.

### 5.3 Targeted therapy: precision intervention in HPV oncogenic pathways

**Figure 3:**
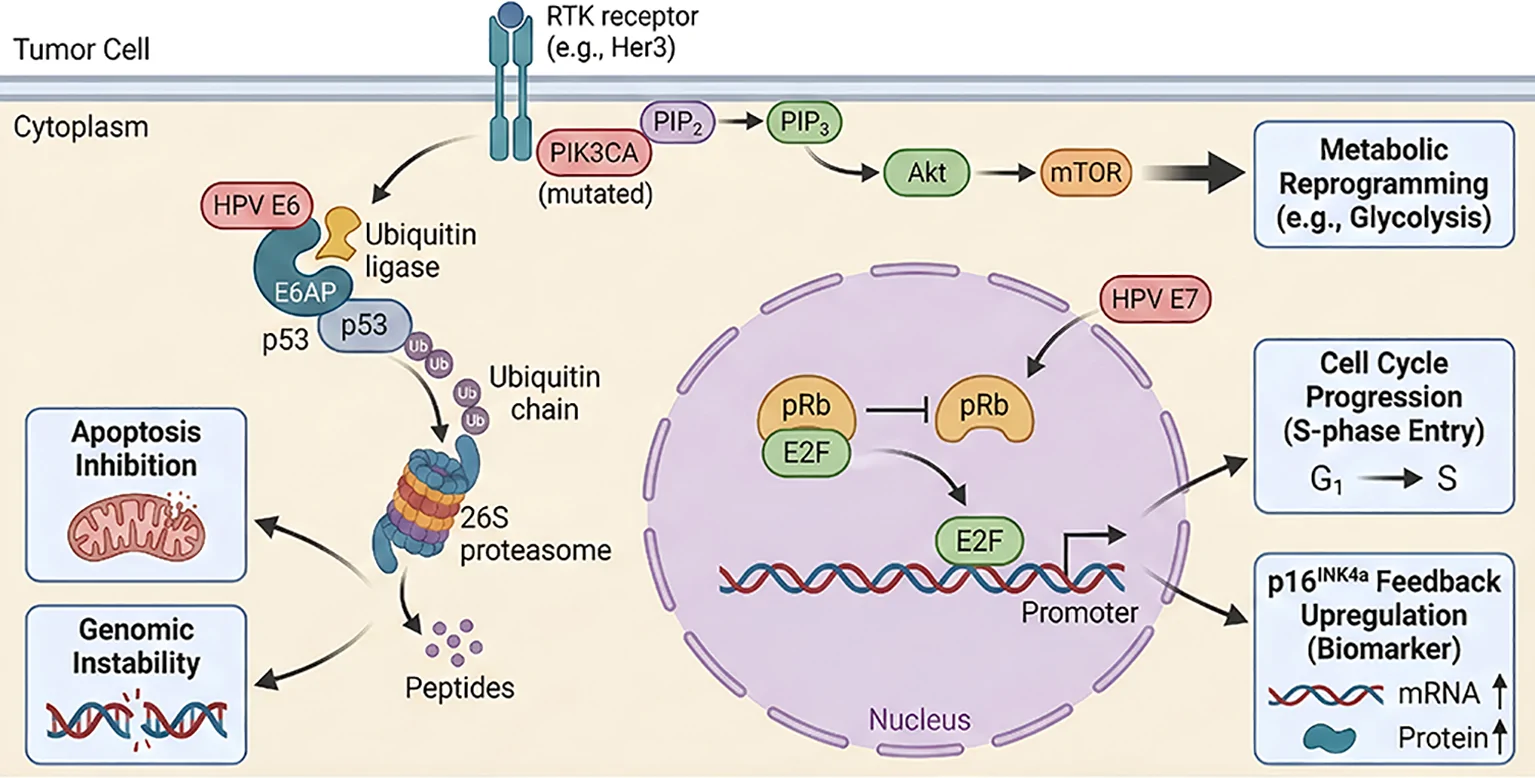
Molecular mechanisms of HPV E6/E7 carcinogenesis. This figure illustrates the core oncogenic mechanisms driven by high-risk HPV oncoproteins. Left (p53 Pathway & Genomic Instability): The HPV E6 oncoprotein recruits the cellular ubiquitin ligase E6AP to form a complex with the tumor suppressor p53, leading to its ubiquitination and degradation via the 26S proteasome. The loss of p53 abrogates apoptosis and impairs DNA repair, resulting in genomic instability. Top (PI3K/Akt/mTOR Pathway): HPV-positive tumors frequently harbor PIK3CA mutations and exhibit upregulated RTK signaling, which activates the PI3K/Akt/mTOR pathway. This cascade promotes metabolic reprogramming and enhances cell proliferation. Right (pRb/E2F Pathway): The HPV E7 oncoprotein binds to and inactivates the pRb, releasing E2F transcription factors to drive aberrant cell cycle entry into the S phase. The inactivation of pRb triggers a compensatory feedback upregulation of p16^INK4a^. While widely utilized as a clinical surrogate biomarker for HPV-associated disease, it is not exclusively specific for transcriptionally active HPV.

HPV-positive OPSCC oncogenesis heavily relies on viral hijacking of host signaling (**Figure 3**). The PIK3CA mutation rate reaches 56%, with PTEN and PI3KR also exhibiting HPV-biased mutations. Furthermore, E6/E7 upregulates Her family expression, forging a Her3-PI3K signaling axis. PIK3CA mutations relentlessly activate the PI3K/Akt/mTOR pathway, upregulating Cyclin D1 (proliferation), phosphorylating Bad (inhibiting apoptosis), and promoting aerobic glycolysis, while Her3 overexpression mediates resistance to PI3K inhibitors.

Crucially, HPV-positive tumors exhibit profound core defects in double-strand break (DSB) repair. In the homologous recombination (HR) pathway, BRCA1/2 and Rad51 are functionally impaired; in the non-homologous end joining (NHEJ) pathway, DNA-PKcs and 53BP1 activities are diminished. Compensatorily, base excision repair (BER) via PARP-1 and microhomology-mediated end joining (MMEJ) are upregulated (88). These DNA damage response (DDR) defects accelerate genomic instability but render tumors exquisitely sensitive to DSB-inducing chemoradiotherapy. Concurrently, the aforementioned viral inactivation of p53 and pRb directly abolishing the G1 checkpoint, while p16 and E2F1 undergo massive amplification. This synergistic inactivation of p53/Rb and E2F1 amplification relentlessly drives S-phase progression. These aberrant PI3K/Akt/mTOR activations, DDR defects, and p53/Rb-E2F1 dysregulations represent distinct molecular vulnerabilities for targeted interventions.

Targeting the PI3K pathway, however, faces significant resistance hurdles. With PIK3CA deletion, AKT is bypassed and reactivated via Skp2, IGF-1R, PDK-1, and mTORC2 (89). PI3Kα inhibitors like Alpelisib frequently fail due to IGF2/IGF1R bypass compensation; silencing IGF2 or adding IGF1R inhibitors (AEW541) profoundly restores anti-tumor efficacy (90). Preclinically, combining the PI3K inhibitor Copanlisib with the pan-ErbB inhibitor Afatinib completely blocked Her family phosphorylation and overwhelmingly induced apoptosis compared to monotherapy (91, 92).

Regarding chemotherapy, HPV inhibits the Fanconi anemia repair pathway by suppressing SERPINB3, inherently enhancing cisplatin cytotoxicity (93). Yet, resistance remains problematic. Importantly, HPV E7-induced HR defects establish an ideal theoretical basis for synthetic lethality via PARP inhibitors (e.g., Olaparib) (94). Epigenetically, HPV upregulates DNMT3B, driving p16^INK4a^ hypermethylation and immune evasion, suggesting DNMT inhibitors could successfully reverse this silencing (95). Thus, combination regimens targeting IGF1R, PARP, and DNMT represent optimal strategies to conquer therapeutic resistance.

While viral-driven DDR defects and PI3K pathway dependencies offer an elegant theoretical rationale for targeted interventions, a glaring translational gap persists. The overarching controversy is the stark disconnect between profound preclinical synthetic lethality and the complete absence of FDA-approved targeted regimens specifically for HPV-positive OPSCC. Currently, identifying robust predictive biomarkers—and safely managing the compounded, overlapping toxicities inherent to multi-target combinations—remain formidable clinical hurdles preventing these molecular strategies from entering the standard-of-care armamentarium.

### 5.4 Controversies in combination strategies and consensus directions

Combination immunotherapies show varying results. A Phase Ib trial of the AMV002 DNA vaccine plus durvalumab demonstrated safety and T-cell induction in recurrent/metastatic disease, yet clinical efficacy remained limited (96). Conversely, lipid nanoparticle HPV mRNA vaccines with ICIs significantly augmented CD8+ T-cell responses preclinically (97). Promisingly, a trial combining the PDS0101 vaccine, PDS01ADC antibody-drug conjugate, and the bispecific antibody bintrafusp alfa achieved acceptable safety and improved OS in advanced, ICI-resistant patients (98).

Furthermore, a Phase II trial of induction chemo-immunotherapy (nivolumab + nab-paclitaxel + carboplatin) followed by response-adaptive locoregional therapy achieved an outstanding 2-year PFS of 90.0% and OS of 91.4% in locally advanced patients (99). Additionally, epigenetic-immune synergies using DNMT inhibitors restore E-cadherin expression suppressed by HPV16 E7, robustly reviving immune cell infiltration and establishing a strong rationale for PD-1 inhibitor combinations (100).

However, post-de-escalation maintenance immunotherapy remains highly controversial. Retrospective data indicate concurrent immuno-radiotherapy (I-RT) yielded significantly worse 3-year OS (81.6% vs. 90.6%) and shortened PFS (HR = 1.69) compared to standard chemoradiotherapy (C-RT), strictly warning that isolated immunotherapy cannot sufficiently control high-risk disease (101). Yet, induction therapies can effectively remodel the tumor microenvironment; for instance, short-term cetuximab induction heavily increases intratumoral CD8+ T-cell infiltration and activates interferon pathways, biologically priming the tumor for sequential PD-1 inhibitors (102). Conversely, the timing of targeted-immune synergy is delicate; the DAHANCA19 trial proved concurrent EGFR inhibition (zalutumumab) and radiotherapy failed to improve survival due to overwhelming cumulative toxicities (103).

In conclusion, the therapeutic landscape for HPV-positive OPSCC is evolving from conventional anatomy-based protocols toward precision interventions centered on virus-host molecular interactions. While de-escalation strategies offer a promising avenue for reducing treatment-related morbidity, their success remains contingent upon moving beyond the limitations of static clinical staging to more refined patient selection. Similarly, the integration of immunotherapy and targeted agents is currently tempered by complex resistance mechanisms and the ongoing search for robust predictive biomarkers. The core of therapeutic innovation likely lies in the strategic reconstruction of treatment sequencing rather than the mere addition of agents. Developing validated multimodal risk stratification systems—integrating genomics, immunology, and dynamic treatment responses—will be essential to transition toward an adaptive model that optimizes the balance between oncological control and functional preservation.

## 6 Prevention and vaccines

### 6.1 The value of HPV vaccines in preventing oropharyngeal cancer

Prophylactic HPV vaccines drastically mitigate HPV-positive OPSCC risk by aggressively preventing persistent high-risk infections. Direct evidence from the U.S. NHANES cohort demonstrated that vaccinated adults (18–33 years) exhibited an 88.2% reduction in oral HPV16/18/6/11 prevalence compared to unvaccinated individuals, with prevalence plummeting to 0% in vaccinated males (104). The U.K. mirrored this profound protective effect, with oropharyngeal HPV16 prevalence falling to 0.5% in vaccinated women versus 5.6% in unvaccinated women; herd immunity correspondingly drove down infection rates among unvaccinated males (105). Long-term U.S. epidemiological models predict that sustaining current male vaccination rates could prevent 792,000 OPSCC cases by 2100; achieving 80% coverage would prevent an additional 142,000 cases (106). Currently, the vaccine-era incidence of HPV-positive OPSCC among young adults is declining, accompanied by significantly improved cancer-specific survival in young men (107). While epidemiological models doubly validate their preventive value, suboptimal global vaccination coverage persists as the core bottleneck restricting population-level benefits.

### 6.2 Breakthroughs in HPV vaccine development

Overcoming tumor immune evasion to establish durable antiviral immunity is the central challenge in therapeutic vaccine development. Recent innovations heavily focus on novel vector systems, adjuvant optimization, and combinatorial strategies. Regarding vectors, bacterial outer membrane vesicles (OMVs) offer intrinsic immunogenicity and robust delivery. Wang et. (108) developed Salmonella OMVs displaying arginine-modified HPV16 E7 (SOMV-9RE7), heavily enhancing antigen cross-presentation to rapidly inhibit tumor growth and recruit massive TILs (108). Additionally, the tetravalent nanovaccine Qβ-HPVag utilizes virus-like particles loaded with toll-like receptor ligands and HPV16 peptides to dynamically activate cDC1/cDC2 subsets and HPV-specific T cells, profoundly hindering murine tumor progression (109). Jia et (110). engineered a sonosensitive polymer (PBAE) that precisely releases tumor antigens and low-dose IL-12 upon ultrasound triggering, robustly activating DCs and amplifying the cytotoxic activity of E7-specific CD8+ T cells (110).

Adjuvant innovations strive to decisively shatter immune tolerance. Rossi et (111). utilized amphiphilic glycosylated lipid A (GLA)—acting as both an inhalability enhancer and built-in adjuvant—to create a dry powder HPV-L2 vaccine that induces robust pulmonary immunity comparable to subcutaneous injection (111). O’Hara et (112). in a preclinical murine model that demonstrated a QS21-adjuvanted vaccine achieved sustained complete regression in >70% of oral HPV tumors by selectively activating HPV-specific CD8+ T cells and NKDC subsets (112).

Combinatorial breakthroughs are particularly compelling. Targeting 16E5 to the DEC-205 dendritic cell receptor, combined with ICIs, generated long-term survival in 70% of tumor-bearing mice (113).Similarly, attenuated LCMV-based HPV16 E6/E7 vaccines combined with anti-PD-1 fully restored anti-tumor efficacy in resistant mice (114). Lee et (115). confirmed that a flagellin-adjuvanted long-peptide vaccine strongly synergized with anti-PD-1 by activating TLR5, decisively suppressing murine tumors (115). Clinically, a Phase Ib/II trial evaluating the TG4001 viral immunotherapy (non-oncogenic HPV16 E6/E7 + IL-2) alongside avelumab in advanced patients showcased excellent response rates and survival benefits in patients without liver metastases, safely inducing potent antigen-specific T-cell responses (116).

The landscape of HPV vaccination is defined by a stark dichotomy between established prophylactic success and ongoing therapeutic challenges. While prophylactic vaccines offer a definitive epidemiological solution to the OPSCC surge, their population-level impact remains severely constrained by suboptimal global coverage, particularly among males. In the therapeutic domain, although novel vectors and adjuvant strategies demonstrate remarkable efficacy in preclinical murine models, translating these robust anti-tumor responses into human trials faces formidable immunological hurdles. Overcoming the profoundly immunosuppressive tumor microenvironment in advanced disease stages and establishing the optimal combinatorial sequencing with immune checkpoint inhibitors remain critical, unresolved clinical imperatives.

To systematically synthesize the evidence levels, clinical benefits, and limitations of different therapeutic strategies for HPV-positive OPSCC, we summarized the representative clinical and preclinical studies worldwide, as shown in **Table 2**. The evidence levels in the table adopt the Oxford 2011 criteria for therapeutic studies.

**Table 2:** Evidence-based summary of primary prevention and therapeutic strategies for HPV-positive OPSCC.

Therapeutic strategy	Specific regimen	Core eligible population	Level of evidence	Core clinical benefit	Major controversies/Limitations	Representative studies
HPV Prophylactic Vaccine	2/4/9-valent HPV prophylactic vaccines	Healthy individuals aged 9–45 years without infection with corresponding vaccine genotypes; adolescents and males are prioritized for vaccination	1a	88.2% reduction in oral infection rates of vaccine genotypes (HPV16/18/6/11) among vaccinated individuals aged 18–33 years; US population models predict prevention of 792,000 male OPSCC cases by 2100	Insufficient global vaccination coverage, with significantly lower rates in males than females; only effective for preventing *de novo* HPV infections, no therapeutic effect on established infections	(104–106)
Surgery-Guided De-Escalation Therapy	TORS/TOS combined with pathology-guided postoperative adjuvant low-dose intensity-modulated radiotherapy (IMRT)	Patients with anatomically resectable HPV-positive early to locally advanced (T1-T2, low N-stage) OPSCC	1b	Excellent perioperative safety; intermediate-risk patients achieve superior short-to-medium-term tumor control with dose-reduced radiotherapy, while significantly preserving swallowing function and quality of life	Surgical indications are strictly limited by tumor anatomical location and surgeon experience; patients with high-risk pathological features (positive margins, extranodal extension) cannot avoid standard-intensity chemoradiotherapy	(65, 66)
Response-Adaptive De-Escalation Therapy	Response-adaptive radiotherapy after induction chemotherapy (Optima paradigm); or hypofractionated definitive radiotherapy alone	p16+/HPV+ OPSCC, covering low-risk to selected high-risk patients	2b	Significantly reduced severe acute and chronic chemoradiotherapy toxicities; selected low-risk patients achieve survival outcomes comparable to standard-intensified therapy	Lack of large multicenter RCT (level 1b) validation; absolute dose threshold for safe de-escalation not established; de-escalation may compromise survival in heavy smokers	(63, 64)
Adoptive Cellular Immunotherapy (TCR-T)	HLA-A*02:01-restricted, engineered TCR-T therapy targeting HPV16 E7_11–19_ peptide	Patients with metastatic/refractory HPV16-positive epithelial cancer carrying the HLA-A*02:01 allele	4	First-in-human demonstration that HPV-specific TCR-T cells can induce objective tumor regression in metastatic epithelial cancers	Eligibility is strictly limited by both viral subtype (only HPV16) and HLA genotype; complex individualized manufacturing process with low accessibility; lack of long-term efficacy data	(73)
Therapeutic Vaccine + Immune Checkpoint Inhibitor	Therapeutic HPV16 vaccines (DNA vaccine AMV002, viral vector vaccine TG4001) combined with PD-L1 inhibitors	HPV16-positive, liver metastasis-free, immune checkpoint inhibitor-naïve patients with recurrent/metastatic OPSCC	4	Confirmed safety and immunogenicity of the combination regimen; induces specific CD8+ T cell expansion; ORR of approximately 23.5% in the recurrent/metastatic setting	High efficacy heterogeneity; only applicable to HPV16-positive patients, with extremely poor efficacy in those with liver metastases; small single-arm studies, lack of head-to-head comparisons	(96, 116)
Targeted Therapy (Monotherapy/Combination)	Dual-pathway combination targeted therapy (ErbB family inhibitor + PI3K inhibitor, PI3K inhibitor + IGF1R inhibitor)	Patients with HPV-positive head and neck squamous cell carcinoma harboring PIK3CA mutations or amplifications	5	Preclinically synergistically inhibits tumor proliferation, induces apoptosis, and reverses monotherapy resistance to PI3K inhibitors	Only preclinical research data; complex bypass resistance mechanisms; lack of human clinical trial validation	(90, 91)
Neoadjuvant Chemoimmunotherapy + Sequential Local Therapy	Neoadjuvant chemoimmunotherapy (nivolumab + nab-paclitaxel + carboplatin) induction, followed by dynamic radiotherapy dose reduction or conversion to surgery based on tumor response	Patients with locally advanced HPV-positive OPSCC	4	86% of patients successfully achieved de-intensification; overall 2-year PFS of 90.0% and OS of 91.4%; responders can safely omit intensified therapy	Optimal induction regimen and duration not standardized; lack of long-term follow-up data; cannot confirm superiority over induction chemotherapy alone	(99)
Therapeutic Vaccine + Immune Checkpoint Inhibitor	Therapeutic HPV16 vaccine combined with PD-1 immune checkpoint inhibitor	HPV16-positive tumors with innate or acquired resistance to single-agent PD-1 inhibitors (only mouse model data)	5	Significantly induces HPV16-specific CD8+ T cell expansion, downregulates exhaustion markers, and achieves >70% complete tumor regression in mouse models	Lack of human clinical trial data; optimal vaccine platform, dosage, and combination timing not established; only targets HPV16 subtype	(112, 114)
De-Escalation Therapy	Concurrent chemoradiotherapy with cetuximab replacing cisplatin	None	1a	No significant benefit; failed to reduce grade 3–5 severe acute/late toxicities, and significantly worsened survival outcomes	3-year OS in the cetuximab group (89.4%) was significantly lower than in the cisplatin group (97.5%); clear clinical contraindication in curative treatment	(68, 69)
Locally Advanced Concurrent Immunoradiotherapy	Concurrent immunoradiotherapy (I-RT) and immune microenvironment assessment	None	2b	Negative OS benefit; trend toward improved prognosis in patients with high PD-L1 expression (CPS≥20) (p=0.07)	Lack of large prospective data; optimal immunotherapy agent and radiotherapy dose not established; survival benefit not confirmed in most populations	(71, 101)

OPSCC, oropharyngeal squamous cell carcinoma; HPV, human papillomavirus; TORS, transoral robotic surgery; TOS, transoral surgery; IMRT, intensity-modulated radiotherapy; TCR-T, T cell receptor-engineered T cell; PD-L1, programmed death-ligand 1; ORR, objective response rate; PFS, progression-free survival; OS, overall survival.

Evidence levels are classified according to the Oxford 2011 criteria: 1a = systematic review of homogeneous randomized controlled trials (RCTs); 1b = individual RCT; 2b = individual cohort study; 4 = case series; 5 = *in vitro*/animal studies. Gray background indicates regimens that are not recommended for routine clinical use due to definitive negative evidence from high-quality trials.

## 7 Conclusion and discussion

### 7.1 Summary and current status analysis

HPV-positive OPSCC constitutes a distinct disease entity with entirely unique molecular profiles, activated immune microenvironments, and remarkably favorable prognoses. Despite rising incidences in Western nations, patients benefit extensively from viral antigen-driven immunity and intact genomic mechanisms (e.g., p53/Rb dysregulation profiles), rendering them highly sensitive to chemoradiotherapy. However, protecting expanding survivor cohorts from severe treatment toxicity without sacrificing cure rates represents the paramount clinical challenge. Consequently, oncological paradigms are transitioning decisively from “one-size-fits-all” standards to biomarker-driven “precision de-escalation”.

### 7.2 Transitioning from static histopathology to dynamic liquid biopsy surveillance

While traditional TNM staging and p16 IHC form the backbone of risk stratification, they consistently fail to predict MRD or early recurrence. Conversely, liquid biopsies evaluating ctDNA and TTMV-HPV DNA are fundamentally reshaping surveillance protocols. Their unmatched sensitivity and specificity position them as independent prognostic biomarkers capable of signaling recurrence months ahead of traditional imaging. When unified with AI radiomics and deep learning algorithms, future diagnostic frameworks will rapidly evolve from static histology to dynamic, real-time multimodal surveillance, enabling highly precise disease tracking and the earliest possible detection of molecular relapse.

### 7.3 Controversies and precision breakthroughs in treatment strategies

Despite the immense appeal of “de-escalation therapy,” current clinical evidence demands extreme caution. Blind de-intensification, such as directly substituting cisplatin with cetuximab, frequently compromises locoregional control and overarching survival benefits. Thus, clinical staging alone is vastly insufficient to isolate true “low-risk” patients. Future therapeutic breakthroughs mandate the strict integration of molecular subtyping and immune characterization to safely guide adaptive treatment interventions. By isolating distinct genomic features and evaluating TIL immune scores, clinicians can accurately delineate optimal de-escalation cohorts. Furthermore, exploiting HPV-driven DDR defects via synthetic lethality and compounding therapeutic E6/E7 vaccines with ICIs represent unparalleled strategies to overcome aggressive therapeutic resistance.

### 7.4 Conclusion and future perspectives

The rigid reliance on static pre-treatment staging must be abandoned in favor of dynamic adaptive risk stratification systems. Liquid biopsies (ctHPV DNA) should be systematically integrated into standard workflows to continually gauge viral clearance kinetics and dictate adaptive dose modulations. Immunotherapy must fiercely pivot toward “antigen specificity”; simple PD-1/PD-L1 blockade yields insufficient returns for many. Emphasizing “precision immunity driven by viral antigens” via advanced therapeutic mRNA/nanocarrier vaccines will decisively breach the immunosuppressive microenvironment. Vitally, de-escalation therapy is strictly inappropriate as a default pathway. Only rigorously defined populations (e.g., low-risk, non-smokers, exceptionally high immune scores) should undergo de-escalation within structured clinical trials. For patients harboring aggressive mutational profiles, targeted combination therapy offers significantly superior safety and efficacy compared to reductive chemoradiotherapy. Ultimately, proactive prevention reigns supreme; aggressively expanding prophylactic HPV vaccination, notably among males, remains the absolute most effective mechanism to establish herd immunity and dismantle the escalating global burden of OPSCC. OPSCC management is officially crossing the threshold into precision medicine. Future endeavors must relentlessly integrate genomics, immunology, and liquid biopsy to architect individualized, full-cycle management paradigms, achieving the paramount dual objectives of maximizing survival and definitively minimizing toxicity.
