# Correction: HPV-specific innovations and controversies in therapeutic strategies for HPV-associated oropharyngeal carcinoma

**DOI:** 10.3389/fimmu.2026.1948255

**Published:** 2026-08-11

**Authors:** Yulun He, Jiaqi Tang, Yun Li, Yating Hu, Yaodong He, Xiaolong Zhang, Yanbing Yao, Jiale Wang, Kunyu Chen, Yuqi Wang, Huan Li, Jianhua Wei

**Affiliations:** State Key Laboratory of Oral & Maxillofacial Reconstruction and Regeneration, National Clinical Research Center for Oral Diseases, Shaanxi Key Laboratory of Stomatology, Department of Oral and Maxillofacial Surgery, School of Stomatology, The Fourth Military Medical University, Xi’an, Shaanxi, China

**Keywords:** HPV-associated oropharyngeal carcinoma, de-escalation therapy, liquid biopsy, immunotherapy, targeted therapy

Affiliation “State Key Laboratory of Oral & Maxillofacial Reconstruction and Regeneration, National Clinical Research Center for Oral Diseases, Shaanxi Key Laboratory of Stomatology, Department of Oral and Maxillofacial Surgery, School of Stomatology, The Fourth Military Medical University, Xi’an, Shaanxi, 710032, China” was omitted from the paper. This affiliation has now been added for author(s) “Yulun He, Jiaqi Tang, Yun Li, Yating Hu, Yaodong He, Xiaolong Zhang, Yanbing Yao, Jiale Wang, Kunyu Chen, Yuqi Wang, Huan Li and Jianhua Wei”.

Affiliation “State Key Laboratory of Military Stomatology and National Clinical Research Center for Oral Diseases, and Shaanxi Clinical Research Center for Oral Diseases, Department of Oral and Maxillofacial Surgery, School of Stomatology, Air Force Medical University, Xi’an, China” was erroneously included in the paper. This affiliation has now been removed for author(s) “Yulun He, Jiaqi Tang, Yun Li, Yating Hu, Yaodong He, Xiaolong Zhang, Yanbing Yao, Jiale Wang, Kunyu Chen, Yuqi Wang, Huan Li and Jianhua Wei”.

All first-level sections following the Introduction were incorrectly typeset as second-level subsections under Section 1. For example, the heading HPV virology basis was published as “1.1 HPV virology basis”, and its subsection HPV life cycle and genotyping was published as “1.1.1 HPV life cycle and genotyping”. This typesetting error applies to all subsequent headings throughout the main text.

A correction has been made to all section headings and the numbering hierarchy of the main text:

The section numbering has been restored to the correct hierarchical structure consistent with the original manuscript:

“1 **Introduction**” remains unchanged as the first-level section. The published “1.1 *HPV virology basis*” is corrected to 2 **HPV virology basis (first-level section)**. The published “1.1.1 *HPV life cycle and enotyping*” is corrected to 2.1 *HPV life cycle and genotyping (second-level subsection under Section* 2). This correction applies to all subsequent first-level and second-level headings across the full main text.

The keywords in the published version were inconsistent with the final confirmed list annotated in the proofreading PDF.

A correction has been made to the section keywords:

Corrected keywords: HPV-associated oropharyngeal carcinoma; De-escalation therapy; Liquid biopsy; Immunotherapy; Targeted therapy

The author contributions statement was erroneously given with non-standard author name formatting. They are should be corrected to:

“Yulun He: Conceptualization, Writing – original draft, Writing – review & editing. Jiaqi Tang: Writing – original draft. Yun Li: Writing – original draft. Yating Hu: Writing – review & editing. Yaodong He: Writing – review & editing. Xiaolong Zhang: Writing – review & editing.Yanbing Yao: Writing – review& editing. Jiale Wang: Writing – review & editing. Kunyu Chen: Writing – review & editing. Yuqi Wang: Writing – review & editing. Huan Li: Conceptualization, Writing – review & editing. Jianhua Wei: Conceptualization, Writing – review & editing, Funding acquisition”.

